# Publisher Correction to: Cognitive Research: Principles and Implications, volume 4

**DOI:** 10.1186/s41235-019-0185-0

**Published:** 2019-08-09

**Authors:** Cognitive Research: Principles and Implications

**Affiliations:** London, UK

**Publisher’s correction to: Cognitive Research: Principles and Implications, volume 4**

An error occurred during the publication of a number of articles in *Cognitive Research: Principles and Implications*. Several articles were published in volume 4 with a duplicate citation number.

In this correction article the old and new citation metadata are published in Table 1.

**Table 1:** Overview of incorrect and correct citation metadata

DOI	Incorrect citation number	Correct citation number
10.1186/s41235-019-0166-3	5	18
10.1186/s41235-019-0167-2	4	17
10.1186/s41235-019-0168-1	1	16
10.1186/s41235-019-0170-7	3	19
10.1186/s41235-019-0172-5	2	20

The original articles have been updated. The publisher apologizes for the inconvenience caused to our authors and readers.

